# The relationship between socioeconomic status, medical accessibility, hope and psychological resilience of caregivers of children with chronic kidney disease in China: structural equation model

**DOI:** 10.1186/s40359-024-01901-8

**Published:** 2024-08-28

**Authors:** Lin Shi, Wenyi Tang, Hengci Zhang, Yunzhi Zhang, Haiping Yang, Daoqi Wu, Xiaoqin Li, Lu Liu, Lijie Liu, Xuelan Chen, Mo Wang

**Affiliations:** 1 Department of Nephrology Children’s Hospital of Chongqing Medical University, National Clinical Research Center for Child Health and Disorders, Ministry of Education Key Laboratory of Child Development and Disorders, Chongqing Key Laboratory of Pediatric Metabolism and Inflammatory Diseases, Chongqing, China; 2 Department of Clinical Data Research, Chongqing Emergency Medical Center, Chongqing Key Laboratory of Emergency Medicine, Chongqing University Central Hospital, Chongqing University, Chongqing, China; 3 Department of Otolaryngology, National Clinical Research Center for Child Health and Disorders, Ministry of Education Key Laboratory of Child Development and Disorders, Chongqing Key Laboratory of Pediatrics, Children’s Hospital of Chongqing Medical University, Chongqing, China; 4 Department of Infectious Diseases of The Second, Affiliated Hospital of Chongqing Medical University, Chongqing, China

**Keywords:** Chronic kidney disease, Caregivers, Socioeconomic status, Medical accessibility, Hope, Psychological resilience, Structural equation model

## Abstract

**Background:**

Chronic kidney disease (CKD) is the third most common cause of death after cancer and heart disease. The continuous treatment of children with CKD was greatly challenged during the COVID-19 pandemic, which significantly impacted the CKD children’s prognosis and the caregivers’ psychological status. However, the influence mechanism of socioeconomic status, medical delay duration, traffic pressure, and online consultation duration on caregivers’ hope and psychological resilience still lacks relevant evidence.

**Methods:**

This study investigated the general social information, hope, and psychological resilience of 247 caregivers with CKD in 13 provinces of China in March 2020. Factor analysis and an exploratory Structural Equation Model ( SEM ) were used to find the best-fit model, and Bootstrapping was used to calculate the 95% CI of indirect effects.

**Results:**

The factor analysis obtained four common factors, namely, socioeconomic status (annual family income, education, and career stability), medical accessibility (online consultation duration, medical delay duration, and traffic pressure), hope (positive attitude, positive action, and intimate relationship) and psychological resilience (optimism, tenacity, and strength), with the cumulative contribution rate of 65.34%. Bootstrapping obtains the socioeconomic status β = 0.30 (95% CI [0.14, 0.47], *P* = 0.002), medical accessibility β = 0.31 (95% CI [0.12, 0.47], *P* = 0.002), and hope β = 0.40 (95% CI [0.27, 0.52], *P* = 0.002) has a direct impact on psychological resilience of CKD children caregivers, followed by medical accessibility β = 0.20 (95% CI [0.10, 0.38], *P* = 0.001) and hope β = 0.23 (95% CI [0.16, 0.32], *P* = 0.001) plays a mediating role between socioeconomic status and psychological resilience. The indirect impact effect β = 0.35 (95% CI [0.25, 0.50], *P* = 0.001) is greater than the direct impact effect β = 0.30 (95% CI [0.14, 0.47], *P* = 0.002).

**Conclusions:**

Sufficient attention should still be given to children with immunodeficiency after the COVID-19 pandemic, such as CKD, to avoid infection of deadly. Secondly, the government should vigorously develop Primary medical institutions to ensure efficient treatment of severe patients in tertiary hospitals; Finally, the professional literacy of medical workers in remote diagnosis and treatment should be improved to enhance the country’s emergency response capacity for similar major public events and the requirements for normalised epidemic prevention and control.

**Supplementary Information:**

The online version contains supplementary material available at 10.1186/s40359-024-01901-8.

## Introduction

Chronic Kidney Disease (CKD) ranks as the third leading cause of death globally, trailing only behind cancer and heart disease [1, 2]. China holds the distinction of having the highest population of CKD patients worldwide. The management of CKD is notably challenging due to its prolonged treatment cycles, the complexity of medication requirements, and the prevalence of multiple complications such as metabolic disorders, infections, and heart failure [3]. Consequently, the mortality rate among children with CKD is thirty times higher than that of their healthy counterparts [4]. The outbreak of the COVID-19 pandemic has significantly compounded the challenges associated with the continuous treatment of chronic diseases. During the pandemic, the United States witnessed a 30% reduction in hospital admissions for renal failure patients due to delayed treatments [5], and the mortality rate from March 1 to August 1, 2020, was 16% higher than anticipated [6]. Concurrently, children with CKD at our centre also experienced delays in receiving medical care and shortages of medication, placing immense psychological pressure on their caregivers.

The Social-Ecological Systems Theory conceptualises the social environment in which individuals live (such as families, groups, or communities) as an ecosystem. It categorises this ecosystem into micro, meso, and macro levels, underscoring these systems’ interconnections and mutual constraints [7]. This theory is increasingly applied in the domains of psychological/behavioural education and chronic disease management for children and adolescents, highlighting the necessity of addressing and optimising the combined influence of multiple factors at the family, community, societal, and policy levels for the healthy development of children.

Psychological Resilience (Resilience), within the Social-Ecological Systems Theory framework, is defined as a process co-facilitated by individuals and their (physical/social) ecology. Research across various fields has corroborated that resilient individuals are more likely to maintain stability and even surpass their pre-trauma functional levels [8]. Distinctively, the focus of resilience research is gradually shifting from the singular perspective of the individual to an interactive adaptation process between individuals and their environments (family, peers, and community) [9, 10].

Hope embodies a vibrant vitality [11], denoting confidence and sentiment in facing adversities, often regarded as a protective factor against negative behaviours [12]. As focal points of positive psychology research, hope and resilience are perceived as wellsprings of inner strength, facilitating individuals to alter their current states and mitigate adverse impacts [13–16]. A perspective suggests that hope can enhance an individual’s resilience through motivation [17].

Medical accessibility (M.A.) refers to the capacity of individuals to obtain appropriate, timely, geographically convenient, and economically affordable healthcare services when needed. It is one of the critical indicators for assessing the fairness and efficiency of a healthcare system within a country or region [18, 19]. At the onset of the pandemic outbreak, the scarcity of medical resources in various locations was directly proportional to the rising density of infections, leading to significant disparities in M.A. This discrepancy resulted in a death rate as high as 3% in the virus’s epicentre, Wuhan City, Hubei Province, compared to an average death rate of only 0.3% and 0% in provinces with relatively abundant per capita medical resources, such as Guangdong and Zhejiang.

Socioeconomic status (SES) refers to an individual or family’s aggregate economic and social position based on income, education, and occupation. Lower SES is associated with poorer housing, diet, working conditions, low income, and social welfare [20]. Moreover, SES is proportionally related to a higher risk of COVID-19 mortality [8]. Studies have shown that educational levels and occupational strata influence the opportunities for individuals to access material resources [21]. In environments of high adversity, individuals with ample resources exhibit stronger resilience [22]. Caregivers of children with CKD face significant economic and psychological challenges due to the excessive caregiving burden [23]. Therefore, it is imperative to assess the relationship between SES and the resilience of vulnerable groups, such as children with chronic illnesses [24, 25].

Preliminary research conducted by our centre suggests that the COVID-19 pandemic has exacerbated social issues such as isolation, scarcity of resources, and psychological fear, which in turn have significantly diminished the level of social support for caregivers of children with CKD compared to a normal control group [26], with a notable increase in anxiety and depression. Among the factors, annual household income emerged as the most critical determinant affecting their psychological burden [27]. The mechanisms through which the SES of CKD children’s caregivers and their resilience interact remain unexplored. Hence, we hypothesise a reciprocal relationship between SES, M.A., hope, and the resilience of caregivers for children with CKD. To test this hypothesis, this paper, set against the backdrop of the COVID-19 pandemic in China, employs a structural equation model to investigate the mechanisms linking the medical plight of children with CKD and the resilience of their caregivers. This inquiry aims to leverage insights for advancing mental health interventions, thereby enhancing the resilience of caregivers for children with chronic illnesses, such as CKD, in the face of significant public health crises.

## Materials and methods

### Study design and recruitment methods

In March 2020, a cross-sectional study was conducted in two comprehensive pediatric hospitals in China’s western region. The research team employed a convenience sampling strategy to collect data from caregivers of children diagnosed with CKD and hospitalised for treatment between January 1, 2016, and November 31, 2019. Initially, caregivers were briefed about the study’s objectives, methodology, and potential risks via telephone, following which verbal consent was obtained. Subsequently, a link to the survey was disseminated through “Wenjuanxing” (the most extensive survey distribution platform in China), and caregivers were guided on completing the questionnaire using electronic devices. Two investigators assessed the quality of the retrieved questionnaires daily, promptly contacting caregivers to verify any missing items or errors detected. Due to the COVID-19 pandemic, interpersonal transmission prevention measures limited researchers to telephone and internet communication with participants. Thus, only verbal informed consent was feasible. The study received approval from the Ethics Committee of Chongqing Medical University Children’s Hospital [2020(9)].

### Subjects

The inclusion criteria of CKD caregivers were (1) compliant with CKD diagnostic criteria [29] and (2) having the ability to use smartphones skillfully. In addition, we excluded participants if their children with CKD: (1) recently received primary mental stimulation, had existing mental disorders or a history of mental illness, (2) had other serious complications such as cancer.

### Training of investigators

Uniform training was provided for five investigators comprised of two nurses (professional titles: nurse or above; educational level: undergraduate or above) and three doctors (professional designations: attending physician or above; academic level: master’s degree or above). The training included: (1) research purpose and methods; (2) questionnaire types, evaluation points, and explanations of items; (3) methods and techniques for collection of questionnaires; and (4) interpretation and signature methods for the informed consent form.

### Data collection

We collected demographic and COVID–19–related data and the resilience and hope scale scores. The collected demographic data included region, diagnosis, gender, and age of children, duration of illness, annual household income, educational background, and occupational stability of caregivers. The collected COVID–19–related data included traffic pressure, delayed treatment duration, online consultation, and stress levels. The minimum acceptable sample size to estimated parameters ratio stands at 5:1, though a ratio of 10:1 is deemed more suitable [28]. Consequently, a sample size ranging from 90 to 180 participants is considered adequate for employing Structural Equation Modeling (SEM) to examine a comprehensive model within a research framework.

### Connor-Davidson Resilience Scale

The study utilised the Chinese version of the Connor-Davidson Resilience Scale (CD-RISC), widely applied across China, to measure resilience [29]. This scale comprises items across three dimensions: tenacity (13 items), strength (8 items), and optimism (4 items), with each item scored from 0 to 4, indicating “not at all,” “rarely,” “sometimes,” “often,” and “almost always,” respectively. The scale’s structure is deemed rational. A total score of 100 points is possible, with higher scores indicating greater resilience. In this study, the Cronbach’s α coefficient for the questionnaire was 0.866, with the dimensions’ Cronbach’s α coefficients being 0.885, 0.753, and 0.797, respectively. The Kaiser-Meyer-Olkin (KMO) measure was 0.913, and Bartlett’s test of sphericity was significant (< 0.001), with the confirmatory factor analysis contributing 48.93% to the total variance.

### Herth Hope Index

The Chinese version of the Herth Hope Index (HHI) [30]was used to measure the level of hope in the current study. The scale contains 12 items divided into positive attitudes towards reality and the future, taking positive actions, and maintaining intimacy with others. Each entry from 1 to 4 means strongly disagree, disagree, agree, and strongly agree. The scale with a total score of 48 points and a higher level of hope indicated a higher score. As a reference, 12–23 points are classified as low levels, 24–35 as intermediate levels, and 36–48 as high levels. In this study, the questionnaire’s Cronbach’s α was 0.849, and each dimension’sα was 0.803, 0.778, and 0.850, respectively. The KMO measure was 0.888, and Bartlett’s test of sphericity was significant (< 0.001), with the confirmatory factor analysis contributing 60.28% to the total variance.

### Statistical methods

Statistical analysis was conducted using SAS9.2 (American Megatrends Inc. 4.6.5) software, and an SEM was constructed using Amos 26.0 (IBM Corporation, Armonk, NY, USA) software.

Firstly, normal tests and homogeneity of variance tests are conducted on continuous variables. Data that do not meet the above conditions are statistically described using M (Q.L., Q.U.), and univariate analysis is undertaken using the Wilcoxon rank sum test. Statistical level: bilateral α = 0.05. Factors with statistically significant results were included in multivariate stepwise linear regression analysis with an inclusion criterion of 0.1 and an exclusion criterion of 0.15. We tested the associations between CD-RISC and HHI using Spearman’s rank correlations. Then, we included the variables screened by the multivariate method in factor analysis to identify potential and observation variables with significant contributions to potential variables. The Kaiser Meyer Olkin (KMO) statistic was greater than 0.7, And Bartlett’s spherical test < 0.05 is used as a condition for factor analysis [31]. On this basis, exploratory SEM [32] was performed to examine all relationships between latent variables and find the best-fit model. The Maximum Likelihood estimation [33] was used for parameter estimation, and the test level was set to α = 0.05. Specifically, a value of chi-square/degrees of freedom ( χ^2^/v) < 3.00, root mean square error of approximation (RMSEA) < 0.08 [34], the goodness of fit index (GFI) > 0.90, comparative fit index (CFI) > 0.90, normalised fit index (NFI) > 0.90, Tucker Lewis index (TLI), and incremental fit index (IFI) > 0.90 [35] can support a good model suitable. Finally, the Bootstrapping algorithm sets the number of replicates to 5000 to calculate the 95% confidence of indirect effects. Interval (CI): The indirect effect is considered significant if 95% CI does not contain zero [36].

## Results

### General demography data

The results indicate that we collected 247 questionnaires from 10 provinces, including Xinjiang, Chongqing, Sichuan, Guizhou, Yunnan, Shanxi, Hubei, Hunan, Zhejiang, and Guangdong, achieving a response rate of 95%. Among the respondents, 83 (33.6%) were freelancers, and 87 (35.22%) were farmers; only 26 individuals (10.53%) possessed a bachelor’s degree or higher, while 127 (51.42%) had an education level of junior middle school or below. Only eight people (3.24%) had an annual income exceeding 150,000 RMB, whereas 111 (44.94%) earned less than 30,000 RMB. Regarding healthcare access, 138 individuals (55.87%) experienced a delay of 1–2 months before seeking medical help, 40 (16.19%) delayed for more than two months, and only 4 (1.62%) did not delay. A total of 109 respondents (44.13%) owned a private car. As for online consultation, only 34 people (13.77%) spent more than 60 min daily, and 61 (24.7%) reported feeling stressed daily. For further details, please refer to Supplementary Table 1.

### Single-factor analysis results

There were statistically significant differences (*P* < 0.01) between the total scores of CD-RISC and HHI regarding occupational stability, education level, annual household income, disease diagnosis, delayed treatment duration, traffic pressure, daily online consultation, and perceived life pressure. The total scores of HHI showed significant differences in age (*P* < 0.05, See Supplementary Table 1).

### Multi-factor analysis results

Optimism, strength, tenacity, positive attitude, intimate relationships, and positive actions were used as dependent variables, and statistically significant factors in univariate analysis were included in the multivariate regression equation. Using multiple stepwise regression analysis to screen variables, it was found that occupational stability is an independent influencing factor for each variable of CD-RISC; education and annual household income are separate influencing factors of optimism; traffic pressure is an independent influencing factor of strength and tenacity of CD-RISC and positive attitude of HHI; daily online consultation is an independent influencing factor on all dependent variables of strength, tenacity, and HHI; delayed treatment duration is an independent influencing factor for all dependent variables of HHI and CD-RISC. The regression coefficients are detailed in Table 1.

**Table 1:** Multi-factor analysis results of caregivers for CKD children(β)

Variable	CD-RISC				HHI			
	Optimism	Strength	Tenacity	Total score	Positive attitude	Intimacy	Positive action	Total score
Region	*	*	*	*	*	*	-0.17**	*
Age	*	*	*	*	*	0.39	*	*
Occupational stability of caregivers	0.30	0.66	1.19	*	*	*	*	*
Educational background	0.58	*	*	*	*	*	*	*
Annual household income	0.59	0.75**	*	*	*	*	*	*
Delayed treatment duration	1.23	2.17	2.80	4.92	0.43	0.75	0.34	1.40
Traffic pressure	*	1.25**	3.42	8.56	0.74	*	*	1.36
Daily online consultation	*	1.37	1.94	4.63	0.36	0.49	0.20	0.99
Feeling life stress	*	*	*	-3.15**	*	-0.53	*	-0.98**

Note: * represents variables not included in the multi-factor model, and * * defines variables included in the initial model but filtered out in the final model; CD-RISC Connor Davidson Resilience Scale; HHI Herth Hope Index

### Spearman correlation analysis results

A correlation analysis was conducted on HHI and CD-RISC, and there was a positive correlation between HHI and various dimensions of CD-RISC (*r* = 0.19–0.58, *P* < 0.01). The remaining results are shown in Table 2.

**Table 2:** Spearman correlation analysis results

CD-RISC/HHI	Positive attitude	Intimacy	Positive action
Optimism	0.38^***^	0.27^***^	0.19^***^
Strength	0.55^***^	0.46^***^	0.38^***^
Tenacity	0.58^***^	0.51^***^	0.38^***^

Note: CD-RISC Connor Davidson Resilience Scale; HHI Herth Hope Index

### Measurement model

We selected 12 numerical variables for factor analysis from the questions related to the response during the pandemic, with a KMO statistic value of 0.873 and Bartlett’s spherical test result < 0.001, indicating that the spherical hypothesis was rejected. Therefore, we used the maximum likelihood estimation to extract four common factors after fitting the variables and named them respectively: SES (annual family income, education, and career stability), M.A. (online consultation duration, medical delay duration, and traffic pressure), HHI (positive attitude, positive action, and intimate relationship) and CD-RISC (optimism, tenacity, and strength), the cumulative contribution rate of all factors are 65.34%. Based on relevant professional knowledge, an initial model was assumed, and maximum likelihood estimation (MLE) was used for SEM statistical testing. Model A is shown in Supplementary Fig. 1, where RMSEA = 0.089. A covariant relationship between e3 and e6 was established based on the maximum correction index (MI) (Model B is shown in Supplementary Fig. 2). RMSEA = 0.078, and the SEM’s fitness index finally reached the standard (fitting index is shown in Table 3), And the factor load of each observation index ranges from 0.67 to 0.91 (*P* < 0.01).

**Table 3:** Fit Index

Fit indicators	Model	χ^2^/v	RMSEA	IFI	NFI	TLI	CFI	GFI
Standard value	-	<3	<0.09	>0.09	>0.09	>0.09	>0.09	>0.09
Fitting value	Model A	2.942	0.089	0.942	0.915	0.922	0.942	0.911
	Model B	2.487	0.078	0.957	0.930	0.940	0.956	0.924

### Structural model

According to Model B in Supplementary Fig. 2, SES, M.A., and HHI can directly affect CD-RISC (see Table 4). We further conducted a mesomeric effect analysis on the model to confirm the relationship between various factors and CD-RISC. The results show that SES and M.A. can also indirectly affect CD-RISC (see Table 5). The overall model explains 70% of the variance. Secondly, the factor load of each measurement path of the model is more than 0.45, and education (*r* = 0.79, *P* < 0.001), online consultation duration (*r* = 0.81, *P* < 0.001), intimacy (*r* = 0.87, *P* < 0.001) and strength (*r* = 0.91, *P* < 0.001) respectively dominate the factors of SES, M.A., HHI, and CD-RISC.

**Table 4:** The correlation coefficient among latent variables

Pathway	Standardised Estimate*	S.E.	C.*R*.
Ses → CD-RISC	0.30	0.14	3.97
M.A. → CD-RISC	0.31	0.38	3.40
HHI → CD-RISC	0.40	0.10	5.74

Note: S.E. Standard Error, C.R. Critical Ratio, Ses Socioeconomic Status, CD-RISC Connor Davidson Resilience Scale, HHI Herth Hope Index, M.A. Medical Accessibility

**Table 5:** Mediating effects of Medical Accessibility and Hope on the relationship between Socioeconomic Status and Psychological Resilience

Pathway	Standardised Estimate*	Effect Ratio (%)	Basic- Corrected 95% CI		*P*
			Lower	Upper	
**Direct Impact**					
SEs→ CD-RISC	0.30	0.46	0.14	0.47	0.002
M.A. → CD-RISC	0.31	0.57	0.12	0.47	0.002
HHI → CD-RISC	0.40	100	0.27	0.52	0.002
**Indirect Impact**					
SEs→ M.A. → CD-RISC	0.20	0.31	0.10	0.38	0.001
SEs→ M.A. → HHI → CD-RISC	0.15	0.23	0.07	0.25	0.002
M.A. → HHI → CD-RISC	0.23	0.43	0.16	0.32	0.001
**Total Impact**					
SEs→ CD-RISC	0.65	100	0.53	0.74	0.002
M.A. → CD-RISC	0.54	100	0.38	0.69	0.002
HHI → CD-RISC	0.40	100	0.27	0.52	0.002

Note: CI confidence interval, Ses Socioeconomic Status, CD-RISC Connor Davidson Resilience Scale, HHI Herth Hope Index, M.A. Medical Accessibility

## Discussion

In the context of the medical challenges faced by children with CKD during the COVID-19 pandemic, this study, grounded in the Social-Ecological Systems Theory, examines the complex interplay of external to internal factors such as the social environment, family environment, and psychological state on the CD-RISC of caregivers. Utilising factor analysis and SEM, this research unveils for the first time the direct impact of the SES of caregivers of children with CKD on their CD-RISC. Additionally, it identifies the mediating roles of M.A. and HHI between SES and CD-RISC, influencing it both directly and indirectly.

### The direct impact of socioeconomic status on psychological resilience

The uneven distribution of medical resources during COVID-19 has become a global public problem [37]. Notably, publications in The Lancet and the Journal of the American Medical Association have highlighted that African Americans, constituting only 30% of the population, account for 70% of COVID-19 mortality rates [38], with lower SES being an independent risk factor influencing these rates [39]. Moreover, a family’s SES can predict the level of CD-RISC in adolescents six years later, with a positive correlation between the two [40]. In our study, a significant proportion of caregivers, 70.03%, were unemployed, 75.71% had not received higher education, and 80.57% had a family annual income below 80,000 yuan. SEM results showed a direct impact of SES on CD-RISC (β = 0.30, 95% CI [0.14, 0.47], *P* = 0.002), accounting for 46% of the total effect. Caregivers with stable jobs, higher education, and incomes, such as government officials, those with bachelor’s degrees or higher, and those with family incomes above 150,000, exhibited stronger CD-RISC. This may be attributed to individuals with higher SES having more comprehensive social support systems, better access to necessary economic and emotional support, facilitating more effective care for CKD children, thereby fostering their own CD-RISC. Hence, the government might consider providing financial assistance or welfare to low-income families to alleviate the economic hardships brought on by the pandemic.

## The indirect influence of socioeconomic status on psychological resilience

### The mediating role of medical accessibility in socioeconomic status and psychological resilience

The study found that because African Americans are more challenged to obtain education and employment opportunities than whites, they face greater economic difficulties. At the same time, they have more difficulty accessing healthcare resources due to the impact of traffic isolation and other factors. Therefore, African Americans have more chronic disease burdens than whites and face higher COVID-19 mortality [41, 42]. Secondly, 84.6% of hospital managers in the United States believe that the treatment effectiveness of non-COVID patients admitted during COVID-19 has deteriorated compared to before due to the delayed treatment time of patients caused by the epidemic [43]. In our study, the indirect effect of SES on CD-RISC through M.A. was significant (β = 0.20, 95% CI [0.10, 0.38], *P* = 0.001), likely because individuals with higher SES can afford safer transportation options and possess better information search and problem-solving skills, enabling them to overcome physical distance barriers to access necessary medical services. With 55.87% of families not owning a private car and public transportation suspended, a significant delay in medical treatment for many children was observed, with 72.06% experiencing delays exceeding one month. Therefore, the government might consider expanding medical insurance coverage to include remote medical services and increasing delivery services for food and medicine to support chronic disease patients who cannot go out. Lastly, public health institutions could disseminate information on virus prevention, chronic disease management, and healthy lifestyles through various channels to better equip chronic disease patients to cope with the pandemic.

### The mediating role of hope in medical accessibility and psychological resilience

The Lancet reported that the pandemic led to a 27.6% increase in global cases of major depressive disorder and a 25.6% increase in anxiety [44] in 2020. Under the shadow of social fear, the uncertainty of treating children’s diseases will reduce the subjective HHI of caregivers [45]. HHI is the protective factor of the COVID-19 dilemma, which directly affects CD-RISC [46, 47]. Secondly, the more COVID-19-related information residents browse weekly, the more confident they are in the face of the epidemic and thus tend to actively prevent and improve outcomes [48]. This study’s results indicate HHI’s indirect impact on M.A. and CD-RISC β = 0.23 (95% CI [0.16, 0.32], *P* = 0.001), accounting for 43% of the total effect. Given that children with CKD are susceptible to COVID-19 [9], only 13.77% of caregivers browse related news for more than 60 min daily. Faced with a high medical treatment delay rate of 98.38% and shortages of supplies, caregivers must confront the dual pressures of bleak treatment prospects and exposure to virus fears, leading to reduced CD-RISC. This finding underscores the importance of HHI as a positive psychological state in maintaining and enhancing the CD-RISC of caregivers for children with CKD under the dual pressures of disease and pandemic. Therefore, healthcare policymakers should prioritise the equitable distribution of medical resources to ensure that patients with chronic conditions receive timely medical services, thereby maintaining and improving the psychological health and quality of life of patients and their families.

### The urgent demand and existing problems of remote medicine in the outbreak of infectious diseases

The COVID-19 pandemic exposed a severe shortage of global medical resources [49]. During this period, online search hot words such as disinfection, isolation, wearing masks, and maintaining social distance showed an exponential growth trend [50]. This study indicates that the duration of online learning is the most important influencing factor for M.A. (*r* = 0.81, *P* < 0.001), possibly due to the current difficulty in implementing the “face-to-face” medical model, which can only obtain information through the internet, and caregivers who are in isolation have more time to browse online information. At present, telemedicine has become a recognised solution, but it seems that it can only be achieved during the sporadic outbreak of the epidemic. From September to December 2022, a larger COVID-19 epidemic broke out in Chongqing [51]. During this period, the government transferred specialised medical personnel from tertiary hospitals to support nucleic acid sampling in grassroots communities and medical work in shelter hospitals [52]. The latter was tired of dealing with hospital infection and treatment of increasing critical patients and had no time to consider Internet hospitals’ operations. At the same time, due to the almost complete paralysis of the express delivery industry, drug delivery is becoming more challenging, leading to significant medical difficulties for chronic disease patients represented by CKD.

The COVID-19 pandemic has ravaged the globe for three years and is set to coexist with humanity long-term. Challenges such as uneven vaccination coverage and virus mutation remain significant global issues. Given the difficulty in predicting virus mutations and pandemic trends, reforming the entire public health system is imperative. Treatment costs for patients with CKD account for 3% of the annual medical budget in developed countries, a significant reason for catastrophic health expenditures [53]. Based on the tertiary prevention theory [54], the government should enhance human resource allocation in primary healthcare institutions to ensure that large tertiary hospitals treat complex and severe patients; secondly, improve the professional competence of medical workers in telemedicine and refine related management standards [55]; finally, establish a comprehensive response mechanism, including prevention measures and medical management, to monitor the pandemic situation and address its long-term health burdens [56].

## Conclusion

This study reveals that during the COVID-19 pandemic, socioeconomic status not only directly affects the psychological resilience of caregivers for children with CKD but also indirectly influences psychological resilience through medical accessibility and hope as mediating variables. This outcome suggests that medical accessibility and hope play a significant role in the relationship between socioeconomic status and psychological resilience, representing a previously undiscovered finding. However, causality cannot be established due to the limitations of cross-sectional studies. Only correlations can be revealed. Additionally, due to time constraints, our study had a small sample size, and online surveys may overlook specific demographics, such as older adults, who are less adept at using smartphones. Therefore, future research should employ various data collection methods to obtain more representative samples and, where appropriate, use longitudinal data to substantiate these conclusions further.

### Electronic supplementary material

Below is the link to the electronic supplementary material.

Supplementary Material 1. Additional file. Supplementary Tables 1- Demographic characteristic and univariate analysis results of caregivers for CKD children (.xlsx).

Supplementary Material 2. Supplementary Fig. 1- Pre-calibration Model A (.tif). Note: SEM path diagram. Ellipses represent potential variables, rectangles represent observed variables, and circles represent residuals. The path between structures represents the direction of causal relationships and coefficients. The higher the coefficient, the more significant the contribution to the variable. SES = Social Economic Status, MA = Medical Accessibility, HHI = Herth Hope Index, CD-RISC = Connor Davidson Resilience Scale, all *P* < 0.001.

Supplementary Material 3. Supplementary Fig. 2- Revised Model B (.tif).

## Data Availability

A statement about the manuscript-The datasets used and/or analysed during the current study are available from the corresponding author on reasonable request.
