# Supplementary figures and images for: The relationship between socioeconomic status, medical accessibility, hope and psychological resilience of caregivers of children with chronic kidney disease in China: structural equation model
Source: BMC Psychol. 2024 Aug 28;12:457. doi: 10.1186/s40359-024-01901-8 (PMC11360847; doi:10.1186/s40359-024-01901-8)

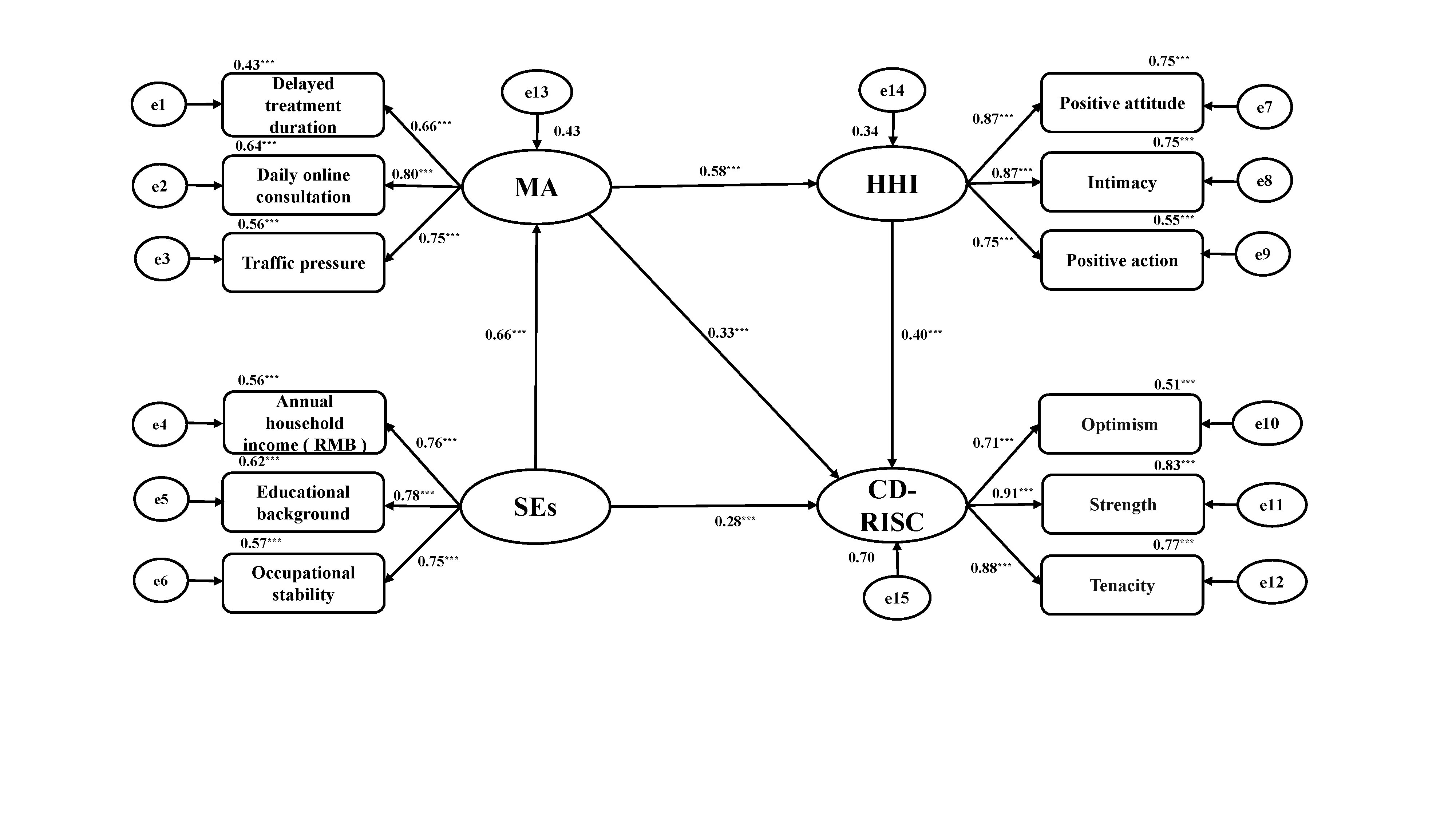

Supplement: Supplementary file 2 — Supplementary Material 2. Supplementary Fig. 1- Pre-calibration Model A (.tif). Note: SEM path diagram. Ellipses represent potential variables, rectangles represent observed variables, and circles represent residuals. The path between structures represents the direction of causal relationships and coefficients. The higher the coefficient, the more significant the contribution to the variable. SES = Social Economic Status, MA = Medical Accessibility, HHI = Herth Hope Index, CD-RISC = Connor Davidson Resilience Scale, all P < 0.001. [file 40359_2024_1901_MOESM2_ESM.tif]

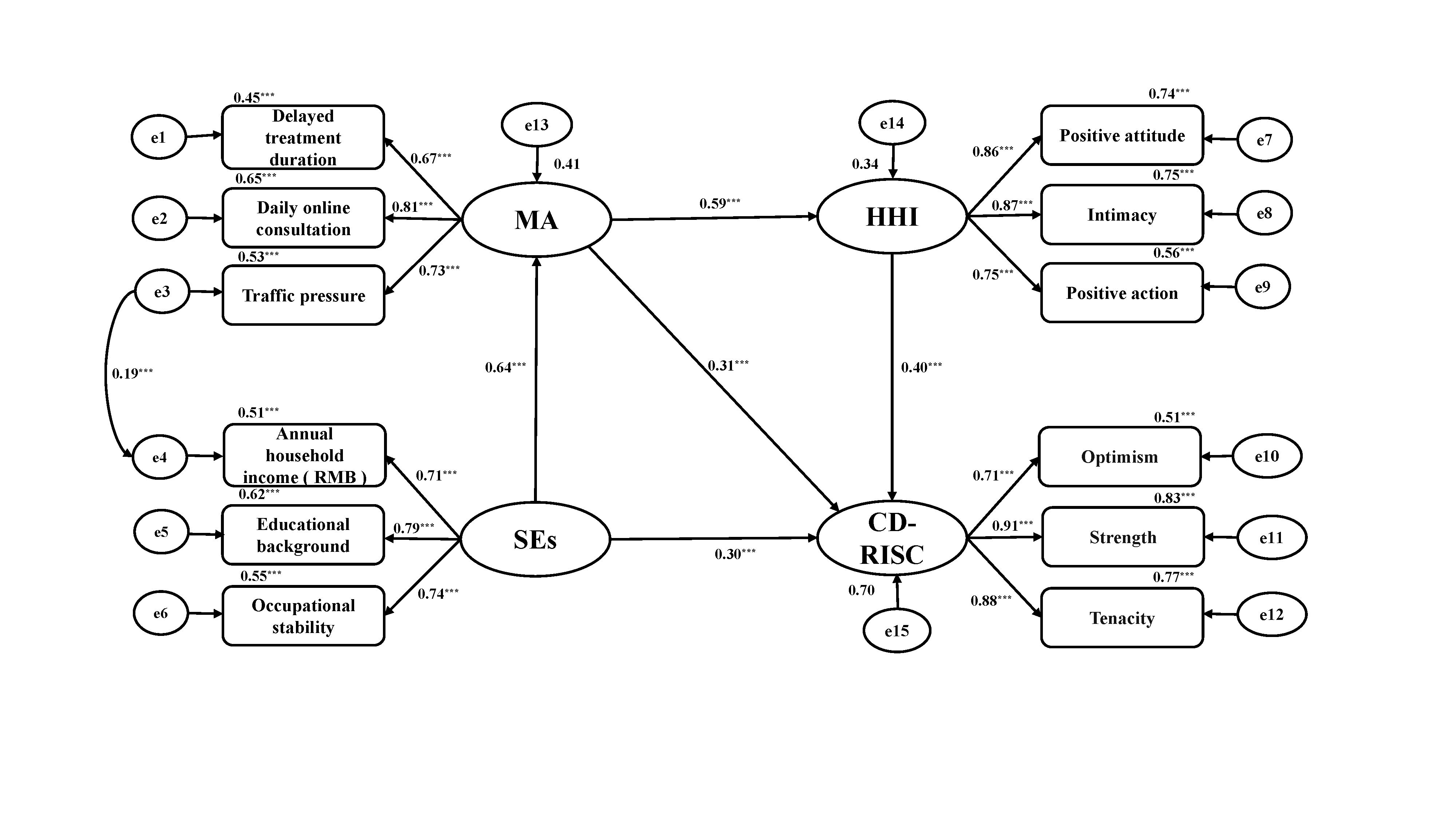

Supplement: Supplementary file 3 — Supplementary Material 3. Supplementary Fig. 2- Revised Model B (.tif). [file 40359_2024_1901_MOESM3_ESM.tif]
